# Could chiropractors screen for adverse drug events in the community? Survey of US chiropractors

**DOI:** 10.1186/1746-1340-18-30

**Published:** 2010-11-17

**Authors:** Monica Smith, Lisa Bero, Lynne Carber

**Affiliations:** 1 Palmer Center for Chiropractic Research, Palmer College of Chiropractic, Davenport, IA, USA; 2 Institute for Health Policy Studies, University of California San Francisco (UCSF), San Francisco, CA, USA; 3 Department of Clinical Pharmacy, University of California San Francisco (UCSF), San Francisco, CA, USA

## Abstract

**Background:**

The "Put Prevention into Practice" campaign of the US Public Health Service (USPHS) was launched with the dissemination of the *Clinician's Handbook of Preventive Services *that recommended standards of clinical care for various prevention activities, including preventive clinical strategies to reduce the risk of adverse drug events. We explored whether nonprescribing clinicians such as chiropractors may contribute to advancing drug safety initiatives by identifying potential adverse drug events in their chiropractic patients, and by bringing suspected adverse drug events to the attention of the prescribing clinicians.

**Methods:**

Mail survey of US chiropractors about their detection of potential adverse drug events in their chiropractic patients.

**Results:**

Over half of responding chiropractors (62%) reported having identified a suspected adverse drug event occurring in one of their chiropractic patients. The severity of suspected drug-related events detected ranged from mild to severe.

**Conclusions:**

Chiropractors or other nonprescribing clinicians may be in a position to detect potential adverse drug events in the community. These detection and reporting mechanisms should be standardized and policies related to clinical case management of suspected adverse drug events occurring in their patients should be developed.

## Introduction

The "Put Prevention into Practice" campaign of the US Public Health Service (USPHS) was launched in 1994 with the dissemination of the *Clinician's Handbook of Preventive Services *that recommended standards of clinical care for various prevention activities. We used the USPHS *Clinician's Handbook of Preventive Services *(1^st ^edition, 1994) as source material to identify a set of prevention activities with potential relevance to chiropractic clinical practice, such as counseling chiropractic patients about physical activity, nutrition, or smoking cessation [1].

The first edition USPHS clinician's handbook identified preventive clinical strategies to reduce the risk of polypharmacy and the attendant risk of overmedication and adverse effects, and outlined for clinicians the basics of polypharmacy counseling and guidelines for simplifying medication regimens [1]. Chiropractors without prescriptive authority would not be directly responsible for simplifying or otherwise altering the prescribed medications regimens of their patients. The overwhelming majority of chiropractors in the U.S. do not prescribe drugs (aside from a small cadre of newly credentialed, limited formulary "advanced practice chiropractors") [2,3]. However, nonprescribing clinicians such as chiropractors may also contribute to advancing drug safety initiatives by identifying potential adverse drug events in their chiropractic patients, and by bringing suspected adverse drug events to the attention of the prescribing clinicians.

## Methods

We conducted a comprehensive multi-topic survey of US chiropractors in 2002-03 to assess their attitudes and behaviors on an encompassing range of various clinical and professional dimensions. All survey questions had been prevalidated by pilot-testing on key informants identified through chiropractic leadership rosters of the Congress of Chiropractic State Associations (COCSA), the Federation of Chiropractic Licensing Boards (FCLB), and the National Board of Chiropractic Examiners (NBCE). Our study methods have been reported in extensive detail elsewhere [4-6]. We drew our randomized survey sampling frame (n = 5,931) from a master list of all US state-board licensed DCs (N = 67,217), and employed 3 mailings plus phone follow-up of non-respondents. Of our mailed surveys to our sampled chiropractors (n = 5,931), we could verify that 2,598 surveys actually reached a valid survey recipient (we deemed 3,333 of our initial list as "invalid" survey attempts, e.g. invalid surveys were returned to us from US Postal Service as "bad address", or we confirmed via followup that surveys were not returned because the DC was retired or deceased, therefore invalid). Of our valid survey contacts, 52% returned their surveys either partially or fully completed. Our overall mail survey response rate of 52% is comparable to that of other surveys of busy professionals [7-11]. We analyzed our survey data using SPSS for Windows version 12.0 (SPSS Inc, Chicago, IL). This study was reviewed and approved by the Palmer College Institutional Review Board.

Reported in this paper, from our 2,598 valid survey contacts, we randomly selected 982 of these surveyed chiropractors to receive a subset of survey questions asking them whether they had ever identified a suspected adverse drug event occurring in one of their chiropractic patients, such as a prescription drug reaction, interaction, or toxicity, and if so, how often this had occurred during the prior two years. We also prompted the chiropractors to describe recent examples in narrative response, such as the chiropractic patient's main reason for seeking the chiropractor's care, what type of adverse drug event the chiropractor potentially identified, what action the chiropractor took or recommended, and the final outcome of the case. Of the 982 chiropractors that received the Adverse Rx Survey Queries, 400 answered the questions, for an item-specific response rate of 41%.

## Results

Of the 400 chiropractors that responded to our Adverse Rx Survey questions, 62% report that they had identified a suspected adverse drug event occurring in one of their chiropractic patients. Over the course of a typical two-year timeframe, half of those chiropractors reported having identified 5 or less adverse drug events in their chiropractic patients, while the other half report identifying upwards of 6 events over a two-year timeframe (see Appendix 1).

Of those surveyed chiropractors who provided additional narrative responses about suspected adverse drug events in their chiropractic patients (see Appendix 2), some chiropractors mentioned specific drugs by name, such as Lipitor (atorvastatin) or Zocor (simvastatin), Celebrex (celecoxib), or Vicodin (hydrocodone). Adverse drug events were also reported by chiropractors in relation to classes of drugs, such as antihypertensive drugs, statins, or NSAIDs (Non-Steroidal Anti-Inflammatory Drugs). The severity of suspected drug-related events detected ranged from mild to severe. Appendix 3 and Appendix 4 typify the narrative responses of chiropractors who offered examples of their experiences with chiropractic patients who had apparent problems while using statins or antihypertensive medications.

## Discussion

### Detection and Reporting

The US Food and Drug Administration (FDA) maintains an internet-based "Medwatch" system for online reporting of adverse reactions and quality problems with drugs, medical devices, other FDA-regulated products such as dietary supplements, cosmetics, medical foods, and infant formulas, or for reporting suspected counterfeit medical products [12]. The user-friendly FDA Medwatch system tracks adverse events reported by consumers and healthcare providers, and adverse event reports may be submitted by more than one reporter. For instance, a single incident of a suspected adverse drug event may be reported by the consumer, their pharmacist, and their physician or other healthcare provider.

Consumers who think that they or someone in their family has experienced a serious reaction to a medical product are encouraged to take the Medwatch reporting form to their prescribing clinician, since that health care provider can provide clinical information from the medical record that can help FDA evaluate the suspected adverse event report. The FDA 3500 Voluntary Adverse Event Report Form can be downloaded directly from the Medwatch website and faxed or mailed to FDA Medwatch, or the adverse event report may be submitted to FDA via telephone (see Appendix 5). Medwatch also advises consumers that health care providers are not required to report to the FDA. If their health care provider chooses not to fill out the FDA Voluntary Adverse Event Report, or if the consumer does not wish to have the form filled out by the health care provider, or if the consumer wishes to also report the suspected adverse event themselves, consumers may contact FDA directly to report the adverse event (see Appendix 5).

Physicians and pharmacists are the healthcare providers who submit reports to FDA Medwatch most frequently [13]. Other healthcare providers include nurses, dentists and others. The MedWatch system is intended to detect safety hazard signals for medical products, in which case the FDA can issue medical product safety alerts or order product recalls, withdrawals, or labeling changes to protect the public health. Important safety information is disseminated to the medical community and the general public via the MedWatch web site and the MedWatch E-list. FDA Form 3500 should be used by healthcare professionals for voluntary reporting of adverse events noted spontaneously in the course of clinical care, not events that occur during clinical trials under an Investigational New Drug (IND) application. The Form FDA 3500 Voluntary Adverse Event Report Form should not be used to report suspected adverse events involving vaccines, vaccine reports should be submitted to the Vaccine Adverse Event Reporting System [14].

Healthcare providers are advised that the Health Insurance Portability and Accountability Act (HIPAA) Privacy Rule specifically permits covered entities (such as hospitals, pharmacists, physicians and other clinicians) to report adverse events and other information related to the quality, effectiveness and safety of FDA-regulated products. This includes reports both to the manufacturers and directly to FDA [15].

FDA Medwatch defines an adverse event as any undesirable experience associated with the use of a medical product in a patient. The event is serious and should be reported when the patient outcome is death, life-threatening, hospitalization (initial or prolonged), congenital anomaly, disability, or the event resulted in a health condition that required medical or surgical intervention to preclude permanent impairment or damage to a patient [16]. For FDA Medwatch reporting purposes, disability is defined as a significant, persistent, or permanent change, impairment, damage or disruption in the patient's body function/structure, physical activities or quality of life. Medwatch advises healthcare providers and consumers to report all suspected serious events, even if they are not certain that the product caused the event.

Serious or fatal adverse drug events reported to the FDA (either directly through the MedWatch program or to drug manufacturers) more than doubled from 1998 through 2005, with reported serious events increasing 4 times faster than the total number of outpatient prescriptions during the period. Among the 15 drugs most frequently named in fatal events, 7 were pain medications [17].

### Clinical Case Management and Coordination of Care

Toward the development of evidence-based best practices for chiropractic clinical case management of suspected adverse drug events in chiropractic patients, the concept of a "triage" approach has been described as a useful aid to the chiropractic clinician [18-20]. During the differential workup of new chiropractic patients, or for new complaints of established patients, triage methods can help to foster safe, efficient, and effective chiropractic patient management. For instance, "red flagging" may identify emergent/urgent cases with signs or symptoms that necessitate immediate or timely referral for medical care. Cautionary "yellow flagging" identifies cases that warrant additional attention and followup, and "green flagging" cases of limited concern.

Older adults are an especially vulnerable population of chiropractic patients at risk of adverse drug events. As noted in the USPHS *Clinician's Handbook of Preventive Services *(1^st ^ed, 1994): "Polypharmacy, the prescribing of multiple drugs for a patient, is most common in older adults because they tend to have more illnesses for which medications are prescribed....Nonprescription drug use among patients over age 65 years is seven times that of the general population. The incidence of adverse drug reactions increases with age and the number of drugs taken. Older adults make many mistakes in taking medications due to deteriorating vision and cognitive function, often with serious consequences....changes in drug metabolism [also] occur with aging. Renal and hepatic function decrease with age, thereby slowing the clearance of medication. Increases in the proportion of body fat and decreases in the proportion of body water that occur with aging lead to an accumulation of fat-soluble medications in adipose tissue and increases in the concentration of hydrophilic medications in the blood." As well, the older chiropractic patient is at higher risk of falling due to medication use and their higher likelihood of having concomitant problems with balance, gait, cognition, vision, strength, postural hypotension, painful arthritis, or depressive symptoms [21-25].

While all elderly patients in the chiropractic practice should be viewed as potential "yellow flag" cases at greater risk of falls, those on medication and in particular frail elderly persons after hospital discharge, are highly vulnerable to falls or other potential problems such as adverse drug reactions, and warrant additional cautionary attentiveness by the prudent clinician [26]. Adverse drug events have been implicated in ED use and hospitalizations that are potentially preventable through better primary care management, particularly for elder patients [27,28]. The medications most commonly involved in adverse reactions among patients seen in the ED were insulins used to treat diabetes, pain medications that contain opiates, and blood thinners. Patients ages 65 and older who experienced adverse reactions to medications were twice as likely to visit EDs and seven times as likely to require hospitalization as younger patients [29].

Younger individuals with a number of comorbidities, or those taking pain medications, may represent another inherently "yellow flag" cautionary population of chiropractic patients. As noted in the USPHS *Clinician's Handbook of Preventive Services *(1^st ^ed, 1994): "Polypharmacy can occur in younger patients as well. As the number of symptoms and diseases increases in an individual, so does the risk of polypharmacy and the attendant risk of overmedication and adverse effects."

Preventable adverse drug events occurring in the ambulatory setting may directly result in serious permanent injury or death [28]. However, even indirect adverse outcomes of drug reactions are important, in that drug reactions such as fatigue, dizziness, or problems with balance may predispose individuals to higher risk of fall, motor vehicle accident, occupational hazard, or other serious indirect consequences [30]. Recently estimated, as many as one fourth to one third of patients may experience an adverse reaction to a medication prescribed in primary care or ambulatory settings [30-32]. Medication reaction symptoms frequently reported include the aforementioned as well as rash, itching, and gastrointestinal problems, and medications that have been typically implicated by patients reporting adverse reactions include oral corticosteroids, nonnarcotic analgesics, and nonsteroidal anti-inflammatory agents (NSAIDS) any of which may be prescribed for back pain [30,31]. Many drug-attributed symptoms occur with every dose and persist for a month or longer, cause patients discomfort and worry, cause patients to seek additional medical attention, compromise patient adherence to prescribed drug regimens, and lower overall patient satisfaction [30-32].

Drug-related mental health effects are a serious concern. For instance, patients undergoing prolonged corticosteroid therapy for inflammatory musculoskeletal or other chronic conditions are at risk for developing symptoms of corticosteroid-induced psychosis [33-37]. Psychiatric symptoms may develop at any time during the course of corticosteroid therapy, or even after cessation of therapy, and may manifest as memory deficits, manic or depressive symptoms, or frank psychosis.

Chiropractic patients using statins may also warrant "yellow flagging". Potential consumer safety issues related to statin use have been identified repeatedly by FDA Medwatch [38-41]. In addition to the FDA warnings about the risk of severe statin-related musculoskeletal affects such as rhabdomyolysis, increasing scientific and clinical attention is being directed toward determining the potential risk for statin-associated tendinous disorders (tendinitis and tendon rupture), and to alerting clinicians that early recognition of such tendinous complications related to statins may be important in preventing serious sequelae in patients being treated with statins [42-44].

Collaborative care is a recognized standard of chiropractic professional practice, and has been defined in chiropractic standards of care as "the reciprocal interprofessional interaction between health care providers in the management of the patient... including basic familiarity with the procedures and terminology of other clinical disciplines... and appropriate and timely referral as needed... including exchange of pertinent information" [45]. Nonprescribing chiropractic clinicians in the US may be customarily taught that "no advice regarding taking or withdrawing from, or increasing or decreasing dosages can be given without violating state scope of practice laws...however when a problem [with medications] is suspected or recognized, it is imperative that the chiropractor either send the patient back to the prescribing [clinician] with informative questions or contact the prescribing [clinician] to discuss the individual patient' [46]. The legal parameters governing chiropractic professional standards of care are largely defined by state rule making authority through administrative boards or direct legislative action (i.e. state scope-of-practice laws), by consensus-based practice guidelines, or by training curricula [47]. However, it is less clear what are the parameters governing cross-disciplinary collaborative care of patients shared between prescribing clinicians and nonprescribing clinicians such as chiropractors. For instance, chiropractors without prescriptive authority may perceive that reporting or referring suspected adverse drug events may place them on questionable grounds in terms of HIPAA, or their scope of practice, or their potential shared exposure to malpractice liability [48]. It is unknown to what extent chiropractors feel that their pregraduate chiropractic training or postgraduate continuing education adequately prepares them to understand the fundamental mechanisms of pharmacology or toxicology, much less how best to apply that understanding toward appropriate detection of suspect cases of adverse drug effects.

Fragmentation and lack of coordination between providers compromises patient safety and undermines quality of care and the efficiency of the health care delivery system [49-52]. Relying on patients to contact other physicians on their own breaks continuity of care [53,54]. Many patients may not report medication-related symptoms to their prescribing clinicians, or prescribing clinicians may not solicit such concerns from the patient nor address those concerns if raised by the patient [55]. Adverse drug events due to patient nonadherence to prescribed drug regimens are potentially preventable by timely identification and notification to prescribing clinicians [56]. Some adverse drug events in primary care, such as medication errors or potential problems with acute medication prescriptions such as narcotics, may also be prevented or averted by pharmacists in the community [57]. Timely detection of adverse drug events is imperative, particularly since certain drug induced problems can be severe or permanently disabling, such as ototoxic loss of hearing or balance [58,59].

In addition to medication intolerance, individual patients may have other reasons for nonadherence, or for being averse to taking prescription medicines themselves or averse to allowing their children to take drugs. For instance individuals or families may have a history of substance abuse, or a suspected familial or genetic predisposition to potential problems with chemical dependency or medication intolerance. In 2006, there were 10.2 million persons aged 12 or older who reported driving under the influence of illicit drugs, and the illicit use category with the largest number of recent initiates to substance use among persons aged 12 or older was the nonmedical use of pain relievers. Among persons aged 12 or older who used pain relievers nonmedically, over half reported that the source of the drug was from a friend or relative for free, and 80% of the time that friend or relative had obtained the drugs from just one doctor [60]. Or individuals may simply prefer not to take drugs, and seek out alternative or complementary non-pharmacologic options for their health care needs. There is a potential role for nonprescribing clinicians such as chiropractors to identify instances of suspected adverse drug reactions or medication intolerance, nonadherence, medication errors, or other problems with prescribed drug regimens.

## Conclusion

Adverse drug events are a major problem and are often undetected. We surveyed US chiropractors about their actual or potential role in advancing patient safety initiatives to monitor adverse drug events. Our findings suggest that chiropractors or other nonprescribing clinicians may be in a position to detect potential adverse drug events in the community. These detection and reporting mechanisms should be standardized and policies related to clinical case management of suspected adverse drug events occurring in chiropractic patients should be developed.

We recommend advancing a multidisciplinary consensus-based approach to improving the integration and coordination of care for patients with suspected adverse drug reactions in the community. Prescribing clinicians, nonprescribing clinicians, and informed others such as pharmacists, should jointly develop and disseminate appropriate standards for intra-disciplinary and inter-disciplinary clinical case management of suspected adverse drug reactions in their shared patients. Optimal collaborative care should include appropriate documentation and communication of useful information, timely notification, and diligent follow-up. More research is needed to better understand the extent, or variation, of chiropractor knowledge, attitudes, and behaviors, regarding their actual and potential roles in screening their patients for potential adverse drug events. More scholarly attention is warranted to further inform expert consensus about what constitutes a useful and necessary skillset (and requisite preparatory training) of nonprescribing clinicians to detect adverse drug events, and to ensure that suspect cases are brought to the attention of the prescribing clinician in a timely and useful manner.

## Competing interests

The authors declare that they have no competing interests.

## Authors' contributions

MS conceived and designed the study, analyzed and interpreted the data, and prepared the manuscript including critical revisions. LB participated in analysis and interpretation of data and in manuscript preparation. LC participated in study design and data acquisition and in manuscript preparation. All authors read and approved the final manuscript.

## Appendix 1: Frequency of chiropractors who identified suspected adverse drug events in their chiropractic patients

*Did chiropractor ever identify a suspected adverse drug event in one of their chiropractic patients, such as prescription drug reaction, interaction, or toxicity?*

Yes   62%

*If Yes, How often did chiropractor identify suspected adverse drug events within the past 2 years?*

5 times or less   48%

6-10 times   19%

11-20 times   15%

21-40 times   10%

>40 times   8%

## Appendix 2: Chiropractors described suspected adverse drug events in their chiropractic patients by Rx drug name, Rx drug type, or other unspecific mention

■ ***Suspected adverse drug events in chiropractic patients with specific mention of the Rx drug name **(e.g. Lipitor, Zocor, Baycol; Celebrex, Vioxx; Prozac, Xanax, Paxil, Wellbutrin, Vicodin, Oxycodone, Percocet, Haldol, Glucophage, Fosomax)*

■ ***Rx mentioned by drug type **(e.g. hypertension meds, blood pressure meds; statins, cholesterol meds; pain meds, narcotics, muscle relaxors, corticosteroids, NSAIDS, anti-inflammatories, prednisone, antidepressants, asthma meds, seizure meds, heart/cardiac meds, coumadin)*

■ ***Other **(e.g. Over-the-Counter (OTC) specifically mentioned by name or type such as NSAIDS, anti-inflammatories, or pain meds; Reactions to Vaccines; Nondescript reference to "meds"; or Vitamins or herbs)*.

## Appendix 3: Example of narrative responses from chiropractors describing suspected adverse drug events in their chiropractic patients using statins

■ "*[chiropractic] patient with multiple severe muscle aches and cramps: resolved within days of removal from zocor/lipitor/baycol, etc*"

■ "*increased low back pain in [chiropractic] patient on lipitor*"

■ "*many patients [in chiropractor's practice] prescribed lipitor med which caused muscle weakness as side effect*"

■ "*several cases [in chiropractor's practice] of cholesterol medication causing systemic pains, [chiropractor] referred cases back to MD who modified meds*"

■ "*several people [in chiropractor's practice] on anticholesterol drugs - when off the drugs did much better overall*"

## Appendix 4: Example of narrative responses from chiropractors describing suspected adverse drug events in their chiropractic patients using antihypertensive drugs

■ "*[chiropractic patient] with fatigue and chest pain [apparent] reaction to new [blood pressure] medication - [chiropractor] referred patient back to prescribing MD...MD changed medication, problem resolved*"

■ "*...[chiropractic patient] with low blood pressure... fainted in my chiropractic office...[patient] was taking high blood pressure med...MD revised patient's medication*..."

■ "*[chiropractic patient] with dizziness, side effect of hypertension med...[chiropractor] recommended patient to consult their primary care provider, MD discontinued med... "*

■ "*many patients [in chiropractor's practice] having problems with blood pressure meds (too much and too little) - chiropractor referred patients back to MD to alter dose*"

■ "*[chiropractic patient] with vertigo, patient on blood pressure Rx... chiropractor referred to prescribing physician for evaluation, changed Rx *"

■ "*chiropractic patient involved in motor vehicle accident, headaches...diagnosed by chiropractor essential hypertension...chiropractor referred patient to primary care for evaluation...patient returned to chiropractor for further care, had adverse reaction to blood pressure meds... chiropractor referred patient back to primary care for re-evaluation [blood pressure med]*"

■ "*[chiropractic] patient with dizziness, taking blood pressure meds...chiropractor sent patient back to cardiologist for meds*"

## Appendix 5: Instructions for Consumer reporting to FDA MedWatch

Reporting to FDA Medwatch System can be conducted online http://www.fda.gov/Safety/MedWatch/default.htm, by phone 1-800-FDA-1088, or by submitting the MedWatch 3500 form by mail (MedWatch 5600 Fishers Lane, Rockville, MD°20852-9787) or fax 1-800-FDA-0178.

The FDA Form 3500 is available for download from FDA Medwatch webpage: http://www.fda.gov/downloads/Safety/MedWatch/HowToReport/DownloadForms/UCM082725.pdf.

Instructions for consumer reporting downloaded on May 13, 2010, from FDA Medwatch webpage: http://www.fda.gov/Safety/MedWatch/HowToReport/ucm053074.htm.

## References

[B1] DHHS/PHS Clinician's Handbook of Preventive Services 1994 (Chap 57, Polypharmacy: pages 319-321).

[B2] CHIROPRACTIC ADVANCED PRACTICE CERTIFICATION REGISTRY. New Mexico Board of Chiropractic Examiners. "Certified advanced practice chiropractic physician" means advanced practice chiropractor who shall have prescriptive authority for therapeutic and diagnostic purposes as authorized by statute and stated by the board in 16.4.15.11 NMAC http://www.nmcpr.state.nm.us/nmac/parts/title16/16.004.0015.htm Accessed Sept 13, 2010.

[B3] Master of Science degree (MS) - Advanced Clinical Practice: An Advanced Degree designed for the Chiropractic Profession. National University of Health Sciences·200 East Roosevelt Road, Lombard, Illinois 60148 http://www.nuhs.edu/show.asp?durki=7 Accessed Sept 13, 2010.

[B4] Smith M Carber LA "Chiropractors as Safety Net Providers: First Report of Findings and Methods from a US Survey of Chiropractors." Journal of Manipulative and Physiological Therapeutics 2007 30 9 718 728 10.1016/j.jmpt.2007.11.001 18082744

[B5] Smith M Carber LA "Survey of US Chiropractor Attitudes and Behaviors about Subluxation." Journal of Chiropractic Humanities 2008 15 19 26

[B6] Smith M Carber LA Survey of US chiropractor perceptions about their clinical role as specialist or generalist Journal of Chiropractic Humanities 2009 16 21 25 10.1016/j.echu.2010.02.009 PMC3342799 22693463

[B7] Asch DA Jedrziewski MK Christakis NA Response rates to mail surveys published in medical journals J Clin Epidemiol 1997 50 1129 36 10.1016/S0895-4356(97)00126-1 9368521

[B8] Sudman S Mail surveys of reluctant professionals Eval Rev 1985 9 349 60 10.1177/0193841X8500900306

[B9] Russell ML Verhoef MJ Injeyan HS McMorland DG Response rates for surveys of chiropractors JMPT 2003 27 43 8 10.1016/j.jmpt.2003.11.005 14739873

[B10] Hay DA A mail survey of health care professionals: analysis of the response J Can Chiropr Assoc 1996 40 162 8

[B11] Cunningham PJ Tu HT A changing picture of uncompensated care Health Aff 1997 16 167 75 10.1377/hlthaff.16.4.167 9248161

[B12] FDA Medwatch System http://www.fda.gov/Safety/MedWatch/default.htm FDA Medwatch WebPage accessed on May 13, 2010.

[B13] AERS reporting to FDA Medwatch by healthcare providers and consumers by year (as of September 30, 2009). Information downloaded on May 13, 2010, from FDA Medwatch http://www.fda.gov/Drugs/GuidanceComplianceRegulatoryInformation/Surveillance/AdverseDrugEffects/ucm070456.htm

[B14] Vaccine Adverse Event Reporting System (VAERS) http://vaers.hhs.gov/index

[B15] HIPAA Compliance for Reporters to FDA MedWatch, Downloaded on May 13, 2010, from FDA Medwatch http://www.fda.gov/Safety/MedWatch/HowToReport/ucm085589.htm

[B16] What is a Serious Adverse Event? Downloaded on May 13, 2010, from FDA Medwatch http://www.fda.gov/Safety/MedWatch/HowToReport/ucm053087.htm

[B17] Moore TJ Cohen MR Furberg CD Serious adverse drug events reported to the Food and drug Administration,1998-2005 Arch Intern Med 2007 167 16 1752 1759 10.1001/archinte.167.16.1752 17846394

[B18] Rubin D Triage and case presentations in a chiropractic pediatric clinic Journal of Chiropractic Medicine 2007 6 94 98 10.1016/j.jcme.2007.05.001 19674702 PMC2647092

[B19] Underwood M Diagnosing acute nonspecific low back pain: Time to lower the red flags? Arthritis & Rheumatism 2009 60 10 2855 2857 10.1002/art.24858 19790072

[B20] Nicholas Henschke N Maher CG Refshauge KM Herbert RD Cumming RG Bleasel J York J Das A McAuley JH Prevalence of and Screening for Serious Spinal Pathology in Patients Presenting to Primary Care Settings With Acute Low Back Pain Arthritis & Rheumatism 2009 60 10 3072 3080 10.1002/art.24853 19790051

[B21] Johnson C Baird R Dougherty PE Globe G Green BN Haneline M Hawk C Injeyan HS Killinger L Kopansky-Giles D Lisi AJ Mior SA Smith M "Chiropractic and Public Health: Current State and Future Vision (Invited editorial)" J Manipulative Physiol Ther 2008 31 397 410 10.1016/j.jmpt.2008.07.001 18722194

[B22] Hawk C Hyland JK Rupert R Colonvega M Hall S Assessment of balance and risk for falls in a sample of community-dwelling adults aged 65 and older Chiropr Osteopat 2006 14 3 10.1186/1746-1340-14-3 16441893 PMC1413542

[B23] The Canadian Chiropractic Association. Don't let a fall get you down http://www.chiropracticcanada.ca/en-us/HealthWellness/PublicEducation/BestFootForward.aspx Accessed May 21, 2010.

[B24] Paniagua MA Malphurs JE Phelan EA Older patients presenting to a county hospital ED after a fall: missed opportunities for prevention Am J Emerg Med 2006 24 413 7 10.1016/j.ajem.2005.12.005 16787797

[B25] Voukelatos A Cumming RG Lord SR Rissel CA Randomized controlled trial of tai chi for the prevention of falls J Am Geriatr Soc 2007 55 1185 91 10.1111/j.1532-5415.2007.01244.x 17661956

[B26] Hanlon JT Pieper CF Hajjar ER Sloane RJ Lindblad CI Ruby CM Schmader KE Incidence and Predictors of All and Preventable Adverse Drug Reactions in Frail Elderly Persons After Hospital Stay Journal of Gerontology 2006 61A 5 511 515 10.1093/gerona/61.5.511 16720750

[B27] Budnitz DS Pollock DA Weidenbach KN National Surveillance of Emergency Department Visits for Outpatient Adverse Drug Events Journal of the American Medical Association 2006 296 15 10.1001/jama.296.15.1858 17047216

[B28] Woods DM Thomas EJ Holl JL Weiss KB Brennan TA Ambulatory care adverse events and preventable adverse events leading to a hospital admission Quality and Safety in Health Care 2007 16 127 131 10.1136/qshc.2006.021147 17403759 PMC2653165

[B29] Budnitz DS National Surveillance of Emergency Department Visits for Outpatient Adverse Drug Events Journal of the American Medical Association 2006 296 15 1858 1866 10.1001/jama.296.15.1858 17047216

[B30] Weingart SN Gandhi TK Seger AC Seger DL Borus J Patient-reported medication symptoms in primary care Arch Intern Med 2004 165 234 240 (2005) 10.1001/archinte.165.2.234 15668373

[B31] Gandhi TK Weingart SN Borus J Seger AC Peterson J Adverse drug events in ambulatory care New England Journal of Medicine 2003 348 1556 64 10.1056/NEJMsa020703 12700376

[B32] Gandhi TK Burstin HR Cook EF Puopolo AL Haas JS Brennan TA Bates DW Drug complications in outpatients J Gen Intern Med 2000 15 149 154 10.1046/j.1525-1497.2000.04199.x 10718894 PMC1495358

[B33] Gagliardi JP Muzyk AJ Holt S When steroids cause psychosis The Rheumatologist 2010 4 10 40 45

[B34] Bolanos SH Khan DA Hanczyc M Assessment of mood states in patients receiving long-term corticosteroid therapy and in controls with patient-rated and clinician-rated scales Ann Allergy Asthma Immunol 2004 92 500 505 10.1016/S1081-1206(10)61756-5 15191017

[B35] Sirois F Steroid psychosis: A review Gen Hosp Psychiatry 2003 25 27 33 10.1016/S0163-8343(02)00241-4 12583925

[B36] Warrington TP Bostwick JM Psychiatric adverse effect of corticosteroids Mayo Clin Proc 2006 81 1361 1367 10.4065/81.10.1361 17036562

[B37] Brown ES Suppes T Khan DA Carmody TJ Mood changes during prednisone bursts in outpatients with asthma J Clinical Psychopharm 2002 22 55 61 10.1097/00004714-200202000-00009 11799343

[B38] Potential Signals of Serious Risks/New Safety Information Identified from the Adverse Event Reporting System (AERS) between April-June 2008. Downloaded on May 13, 2010, from FDA Medwatch http://www.fda.gov/Drugs/GuidanceComplianceRegulatoryInformation/Surveillance/AdverseDrugEffects/ucm085916.htm

[B39] FDA Drug Safety Newsletter - Volume 1, Number 4, Summer 2008. This publication provides postmarketing information to healthcare professionals to enhance communication of new drug safety information, raise awareness of reported adverse events, and stimulate additional adverse event reporting. Reports of rhabdomyolysis associated with the concomitant use of amiodarone (marketed as CORDARONE and PACERONE) and simvastatin (marketed as ZOCOR and generics) or simvastatin-combination products (marketed as VYTORIN and SIMCOR) downloaded on May 13, 2010, from the FDA Medwatch http://www.fda.gov/Drugs/DrugSafety/DrugSafetyNewsletter/ucm109176.htm

[B40] Serious Muscle Injury with Simvastatin/Amiodarone Combination. FDA Patient Safety News, November 2008. Downloaded from FDA Medwatch http://www.accessdata.fda.gov/psn/transcript.cfm?show=81

[B41] Potential Signals of Serious Risks/New Safety Information Identified from the Adverse Event Reporting System (AERS) between July - September 2009. Downloaded on May 13, 2010, from FDA Medwatch http://www.fda.gov/Drugs/GuidanceComplianceRegulatoryInformation/Surveillance/AdverseDrugEffects/ucm199544.htm

[B42] Marie I Delafenetre H Massy N Thuillez C Noblet C the Network of the French Pharmacovigilance Centers Tendinous disorders attributed to statins: a study on ninety-six spontaneous reports in the period 1990-2005 and review of the literature Arthritis & Rheumatism (Arthritis Care & Research) 2008 59 3 367 372 10.1002/art.23309 18311771

[B43] Pullatt RC Gadarla MR Karas RH Alsheikh-Ali AA Thompson PD Tendon rupture associated with simvastatin/ezetimibe therapy Am J Cardiol 2007 100 152 3 10.1016/j.amjcard.2007.02.068 17599460

[B44] Tendon rupture and statin therapy: is there a link? Comments on the article by Marie et al 10.1002/art.23917 18668581

[B45] Haldeman S Chapman-Smith D Petersen DM Guidelines for Chiropractic Quality Assurance and Practice Parameters 1993 Aspen Publishers, Inc, Gaithersburg, MD 161 166 ISBN: 0-8342-03758.

[B46] Souza TA Differential Diagnosis and Management for the Chiropractor 2009 4 Jones and Bartlett Publishers, Sudbury Massachusetts 1076 ISBN-13: 978-0-7637-5282-8

[B47] Ladenheim CJ Sherman RP Sportelli L Professional Chiropractic Practices: Ethics, Business, Jurisprudence and Risk Management 2001 PracticeMakers Products, Inc., PO Box 213, 175 Delaware Avenue, Palmerton, PA 18071 161 165 ISBN # 0-9703839-1-6.

[B48] Rothschild JM Federico FA Gandhi TK Kaushal R Williams DH Bates DW Analysis of Medication-Related Malpractice Claims: Causes, Preventability, and Costs Arch Intern Med 2002 162 2414 2420 10.1001/archinte.162.21.2414 12437399

[B49] Greene BR Smith M Haas M Allareddy V "How Often Are Physicians and Chiropractors Provided With Patient Information When Accepting Referrals?" Journal of Ambulatory Care Management 2007 30 4 344 346 17873666 10.1097/01.JAC.0000290403.89284.e0

[B50] Allareddy V Greene BR Smith M Haas M Liao J "Facilitators and Barriers to Improving Interprofessional Referral Relationships Between Primary Care Physicians and Chiropractors." Journal of Ambulatory Care Management 2007 30 4 347 354 17873667 10.1097/01.JAC.0000290404.96907.e3

[B51] Smith M Greene BR Haas M Allareddy V "Intra-professional and inter-professional referral patterns of chiropractors." BMC Chiropractic and Osteopathy 2006 14 12 10.1186/1746-1340-14-12 PMC1552072 16824214

[B52] Greene B Smith M Allareddy V Haas M "Referral Patterns and Attitudes of Primary Care Physicians Towards Chiropractors." BMC Complementary and Alternative Medicine 2006 6 5 16509963 10.1186/1472-6882-6-5 PMC1456998

[B53] Lee T Pappius EM Goldman L Impact of inter-physician communication on the effectiveness of medical consultations Am J Med 1983 74 1 106 112 10.1016/0002-9343(83)91126-9 6849320

[B54] Mainous AG Gill JM Zoller JS Wolman MG Fragmentation of patient care between chiropractors and family physicians Arch Fam Med 2000 9 5 446 450 10.1001/archfami.9.5.446 10810950

[B55] Weingart SN Gandhi TK Seger AC Seger DL Borus J Burdick E Leape LL Bates DW Patient-Reported Medication Symptoms in Primary Care Arch Intern Med 2005 165 234 240 10.1001/archinte.165.2.234 15668373

[B56] Gurwitz JH Field TS Harrold LR Incidence and preventability of adverse drug events among older persons in the ambulatory setting JAMA 2003 289 1107 1116 10.1001/jama.289.9.1107 12622580

[B57] Hansen LB Fernald D Araya-Guerra R Westfall JM West D Pace W Pharmacy clarification of prescriptions ordered in primary care: A report from the applied strategies for improving patient safety (ASIPS) collaborative J Am Board Fam Med 2006 19 1 24 30 10.3122/jabfm.19.1.24 16492002

[B58] Schacht, Jochen and Talaska, Andra (2008, June 26. Kresge Hearing Research Institute, University of Michigan, Ann Arbor MI, USA). Ototoxicity - Drug-induced Loss of Hearing and Balance SciTopics http://www.scitopics.com/Ototoxicity_Drug_induced_Loss_of_Hearing_and_Balance.html Retrieved September 8, 2010

[B59] Mudd PA Edmunds AL Glatz FR Campbell KCM Rybak LP Inner Ear, Ototoxicity http://emedicine.medscape.com/article/857679-overview (Updated Jul 1, 2010). Retrieved Sept 8,2010.

[B60] Substance Abuse and Mental Health Services Administration (2007). Results from the 2006 National Survey on Drug Use and Health: National Findings (Office of Applied Studies, NSDUH Series H-32, DHHS Publication No. SMA 07-4293) Rockville, MD

